# DRAM1 Promotes Lysosomal Delivery of *Mycobacterium marinum* in Macrophages

**DOI:** 10.3390/cells12060828

**Published:** 2023-03-07

**Authors:** Adrianna Banducci-Karp, Jiajun Xie, Sem A. G. Engels, Christos Sarantaris, Patrick van Hage, Monica Varela, Annemarie H. Meijer, Michiel van der Vaart

**Affiliations:** Institute of Biology Leiden, Leiden University, Einsteinweg 55, 2333 CC Leiden, The Netherlands

**Keywords:** DRAM1, innate immunity, lysosomes, macrophages, mycobacterium, phagosomes, vesicle trafficking, xenophagy

## Abstract

Damage-Regulated Autophagy Modulator 1 (DRAM1) is an infection-inducible membrane protein, whose function in the immune response is incompletely understood. Based on previous results in a zebrafish infection model, we have proposed that DRAM1 is a host resistance factor against intracellular mycobacterial infection. To gain insight into the cellular processes underlying DRAM1-mediated host defence, here we studied the interaction of DRAM1 with *Mycobacterium marinum* in murine RAW264.7 macrophages. We found that, shortly after phagocytosis, DRAM1 localised in a punctate pattern to mycobacteria, which gradually progressed to full DRAM1 envelopment of the bacteria. Within the same time frame, DRAM1-positive mycobacteria colocalised with the LC3 marker for autophagosomes and LysoTracker and LAMP1 markers for (endo)lysosomes. Knockdown analysis revealed that DRAM1 is required for the recruitment of LC3 and for the acidification of mycobacteria-containing vesicles. A reduction in the presence of LAMP1 further suggested reduced fusion of lysosomes with mycobacteria-containing vesicles. Finally, we show that DRAM1 knockdown impairs the ability of macrophages to defend against mycobacterial infection. Together, these results support that DRAM1 promotes the trafficking of mycobacteria through the degradative (auto)phagolysosomal pathway. Considering its prominent effect on host resistance to intracellular infection, DRAM1 is a promising target for therapeutic modulation of the microbicidal capacity of macrophages.

## 1. Introduction

Innate immune cells, including macrophages, are the gatekeepers of the immune system; they ingest microbes into internal compartments called phagosomes, they mount a range of cell-autonomous defence responses, and they alert other components of the immune system. A key cell-autonomous defence response is the phagolysosomal pathway, which delivers microbes through a phagosome maturation process to lysosomes. Upon lysosomal fusion, microbes are exposed to an acidic environment, antimicrobial peptides, and degradative enzymes [1]. However, several intracellular pathogens are able to withstand the phagocyte defences because they are able to inhibit the maturation of phagosomes, adapt to the hostile environment of lysosomes, or breach the integrity of the phagosome membrane and invade the cytosol [2]. Invasion of the cytosol exposes these pathogens to a secondary innate immune defence process, termed autophagy, which serves as a mechanism to preserve cellular homeostasis [3]. In the anti-microbial autophagy response, also known as xenophagy, selective autophagy receptors recognise microbes marked for proteasomal degradation by ubiquitin coating [4]. These receptors then recruit the autophagy machinery through interaction with microtubule-associated proteins 1 light chain 3 (hereafter LC3). As a result, microbes are captured inside double-membrane vesicles, called autophagosomes, which subsequently fuse with lysosomes to degrade their contents. LC3 may also be conjugated to the single membrane of phagosomes, in an autophagy-related process known as LC3-associated phagocytosis (LAP), which is thought to enhance the subsequent phagolysosomal degradation process [5]. Several pathogens have evolved mechanisms to inhibit phagolysosomal degradation, LAP or the autolysosomal route at various points in these pathways [6,7].

The genus *Mycobacterium* comprises several human pathogens that can subvert both the phagolysosomal and autolysosomal pathways, in order to facilitate their intracellular survival in macrophages [8,9,10,11]. The most notorious mycobacterial species is *Mycobacterium tuberculosis* (*Mtb*), which causes 10 million cases of tuberculosis (TB) and close to 1.5 million deaths every year [12]. However, the medical importance of mycobacteria extends beyond *Mtb* to a large collection of nontuberculous mycobacteria (NTM). For example, *Mycobacterium avium* lung infections are associated with poor clinical outcomes, and are notably on the rise [13,14]. Another example of NTMs is *Mycobacterium marinum* (*Mm*), known for outbreaks of a TB-like infection in fish in aquacultures, as well as for skin infections in humans [15]. *Mm* is closely related to *Mtb*, sharing 3000 orthologous proteins with an average amino acid identity of 85% [16]. This similarity includes the RD1 virulence locus, encoding the type VII secretion system ESX-1 and its secreted proteins, ESAT-6 and CFP-10 [17]. In both *Mm* and *Mtb*, the bacteria require the ESX-1 system to evade phagolysosomal degradation and invade the cytosol [18]). As a countermeasure, ESX-1-competent *Mm* or *Mtb* are targeted by xenophagy [19,20,21]. However, it remains unknown as to what extent xenophagy provides host protection during clinical infection, as several studies report that mycobacteria possess virulence mechanisms to inhibit autophagy-mediated degradation [22]. Likewise, *Mtb* has been shown to inhibit the autophagy-related LAP pathway [23].

DRAM1, standing for damage-regulated autophagy modulator 1, is the founding member of a family of five stress-inducible autophagy regulators [24,25]. DRAM1 functions as a target of p53 signalling in the UV-damage response and as a target of NF-κB signalling during *Mm* and *Mtb* infection [20,24]. In addition, DRAM2 has been implicated in defence against mycobacteria, whereas DRAM3, 4, and 5 have not been linked to infectious diseases [26,27,28,29]. We have shown previously that DRAM1 provides a link between pathogen recognition and the activation of autophagy, as its induction during mycobacterial infection was found to be dependent on MyD88 and NFκB, central mediators of the Toll-like receptor pathway [20]. Using a zebrafish model for *Mm* infection, we showed that knockdown or mutation of the zebrafish ortholog of DRAM1 causes a hyper-susceptibility to infection, while its overexpression increases host resistance against *Mm* [20,30]. We also obtained evidence that the zebrafish DRAM1 ortholog promotes the association of *Mm* with LC3 and increases the lysosomal acidification of *Mm*-containing compartments [20,30]. However, due to the lack of antibody tools for zebrafish, we have not been able to study how DRAM1 colocalises with mycobacteria, autophagosomes and lysosomes during the course of infection.

To study DRAM1 localisation and function during mycobacterial infection in a relevant mammalian cell type, we used the mouse RAW264.7 macrophage cell line, which has been frequently employed for *Mtb* autophagy studies [31,32,33]. *Mm* infection of this cell line leads to rapid permeabilisation of phagosomes and subsequent ubiquitination of bacteria, marking them as targets for xenophagy [34]. Here, we show the progression of the colocalisation of DRAM1 with *Mm* over the course of infection, and study this in relation to LC3, LysoTracker and LAMP1 patterns, as markers for autophagosomes and (endo)lysosomes. In addition, we generated DRAM1 knockdown macrophage cell lines to show that DRAM1 knockdown reduces the trafficking of LC3 to *Mm*, as well as reduces the acidification of *Mm*-containing vesicles. Furthermore, upon DRAM1 knockdown, the *Mm* infection rate increased. These results support the central role of DRAM1 in host resistance to intracellular infection, by promoting the trafficking of mycobacteria in the (auto)phagolysosomal pathway.

## 2. Materials and Methods

### 2.1. Macrophage Cell Culture

The RAW 264.7 macrophage cell line was maintained in Dulbecco’s Modified Eagle’s Medium (DMEM) with high glucose (Sigma-Aldrich, Zwijndrecht, the Netherlands, D6546), with 10% fetal calf serum (FCS) (Gibco, Loughborough, UK, F2442) at 37 °C in a 5% CO_2_ atmosphere. The day before *Mm* infection, RAW 264.7 cells were seeded on sterile coverslips inserted in 12-well plates (2.5 × 10^5^ cells/well for the 0–150 mpi *Mm* infection experiments or 4 × 10^5^ cells/well for the *Mm* infection rate experiment) and incubated overnight at 37 °C with 5% CO_2_.

### 2.2. Mm Culture and Infection Experiments

The day before cell infection, *Mm* M-strain, fluorescently labelled with mWasabi [35], was cultured in Difco Middlebrook 7H9 medium (Becton Dickinson, Vianen, the Netherlands, BD271310) with 10% BBL™ Middlebrook albumin–dextrose–catalase (Becton Dickinson, 211887) and 50 µg/mL hygromycin (Sigma-Aldrich, SC-506168A), at 28.5 °C in a static incubator. On the day of infection, *Mm* was washed twice with PBS, and optical density (OD) was measured at 600 nm. An OD 600 nm value of 1 is equal to 10^8^ *Mm*/mL [36]. Wild-type RAW 264.7 cells were seeded in order to investigate the colocalisation of DRAM1, LC3 and LysoTracker with *Mm*, while DRAM1 knockdown and scramble control cell lines were used for the functional analysis of DRAM1. Cells were then infected with a multiplicity of infection (MOI) of 10, immediately centrifuged at 500× *g* for 10 min to synchronise infection, as described in [37], and incubated at 32 °C with 5% CO_2_. After 30 min, cells were washed twice with PBS to remove extracellular *Mm* and then incubated with LysoTracker-enriched medium for up to 150 min at 32 °C to obtain samples for immunofluorescence at 30 min intervals. At each 30 min interval, cells were fixed with Pierce™ 4% formaldehyde (PFA) (Thermo Scientific, Loughborough, UK, 28908) for 20 min at room temperature (RT). To analyse infection rates, cells were infected at a MOI of 10 without centrifugation and incubated at 32 °C. After 1 h, cells were either fixed with PFA or washed with PBS twice, in order to remove extracellular bacteria, and were then incubated with fresh medium for 6 h.

### 2.3. Immunofluorescence

After PFA fixation, cells were washed with PBS and permeabilised with 0.2% Saponin (Sigma-Aldrich, 47036) in PBS for 15 min, before blocking with 1% bovine serum albumin (BSA) (Sigma-Aldrich, A4503) in PBS for 1 h at RT. Next, cells were incubated with the appropriate primary antibody, specifically DRAM1 (1:500) (Invitrogen, Loughborough, UK, OSD00007G), LC3 (1:500) (Novus Biologicals, Cambridge, UK, NB100-2331), or LAMP1 (1:1000) (Abcam, Cambridge, UK, ab24170) for 1 h at RT. All primary antibodies were tested for a lack of cross-reaction with in vitro-cultured *Mm* (Supplementary Figure S1). For single immunostaining, goat-anti-rabbit Alexa Fluor™ Plus 647 (1:1000) (Invitrogen, A-21245) was added to detect the primary antibodies. Controls for antibody specificity (secondary antibodies in the absence of primary antibodies) were all negative. Between each step, cells were washed four times with PBS for 5 min each. For LC3 and ubiquitin double immunostaining, LC3 rabbit antibody and mono- and poly-ubiquitinylated conjugates monoclonal mouse antibody (1:1000) (Enzo Life Sciences, Zandhoven, Belgium, BML-PW8810-0100) were incubated simultaneously for 1 h at RT. Goat-anti-rabbit Alexa Fluor™ Plus 647 (1:1000) (Invitrogen, A-21245) and goat anti-mouse Alexa Fluor™ Plus 488 (1:1000) (Invitrogen, A32723) were added to detect the primary antibodies. For DRAM1 and LC3 double immunostaining, cells were incubated with DRAM1 primary antibody (1:250) for 2 h and then washed with PBS 3 × 10 min. Then, the goat-anti-rabbit Fab 647 (1:50) (Sanbio, Uden, the Netherlands, 111-607-003) was incubated overnight. The next day, cells were washed 6 × 10 min. Cells were incubated with Fab secondary antibody again for 2 h at RT. After washing, cells were incubated with the LC3 primary antibody (1:250) for 1.5 h, and afterwards washed for 3 × 10 min. Lastly, cells were incubated with the second secondary antibody goat-anti-rabbit Alexa Fluor™ 568 (1:1000) (Invitrogen, A-11011) for 2 h at RT, and then washed for 4 × 5 min. Cover slips were mounted onto glass slides with Prolong Gold Antifade Reagent (Invitrogen, P36966) for imaging.

### 2.4. LysoTracker Labelling

Acidic vesicles were visualised by incubating RAW 264.7 cells in medium that was enriched with LysoTracker™ Red DND-99 (1:2500) (Invitrogen, L7528) for at least 1 h prior to fixation at 37 °C [38]. For non-infected cells, LysoTracker staining was performed for 30 min before fixation.

### 2.5. DRAM1 shRNA Knockdown

To knockdown DRAM1, three short hairpin RNAs (shRNAs) against DRAM1 (NM_027878) from the Mission library (Sigma-Aldrich) were used, with scrambled shRNA as a control. pLKO.1-puro shRNA constructs were transduced into RAW 264.7 cells. The day before transduction, 1 × 10^6^ RAW 264.7 cells were seeded per T25 flask in DMEM-high glucose with 10% FCS and incubated overnight at 37 °C with 5% CO_2_. Then, 24 h later, cells were transduced at an MOI of 4 or 8, supplemented with 8 µg/mL polybrene (Sigma-Aldrich, TR1003), and incubated at 37 °C with 5% CO_2_. The medium was refreshed 24 h after transduction. After 48 h of transduction, 3 µg/mL puromycin (Gibco, A1113803) was added. Every 2–3 days, the medium was replaced until cells were 80–90% confluent. To isolate single clones with successful DRAM1 knockdown, cells from each condition were diluted in DMEM-high glucose with 10% FCS and 3 µg/mL puromycin to achieve 1 cell/well in a 96 well plate. The following day, wells containing 1 cell were marked. Once marked wells reached 80–90% confluency, cells were transferred to 12-well plates and subsequently to T25 flasks. Antibiotic pressure was removed from the medium to confirm the stability of DRAM1 shRNA integration into cells.

### 2.6. Quantitative PCR

Cell pellets containing 1 × 10^6^ cells were dissolved in TRIzol (Invitrogen, 15596018) and then chloroform (Supelco^®^, Loughborough, UK, 1.07024.2500). After centrifuging at 12,000× *g* for 15 min at 4 °C, the aqueous phase containing the RNA was transferred to a new tube. One volume of 70% ethanol was added and then transferred to a column from the RNeasy mini kit (Qiagen, Hilden, Germany, 74104). RNA isolation was performed according to the manufacturer’s protocol, which included on-column DNAse digestion. A total of 500 ng of each RNA sample, measured with the Nanodrop (Thermofisher), was converted to cDNA with the C1000 Touch Thermal Cycler, using the iScript cDNA Synthesis Kit according to the manufacturer’s protocol (Bio-Rad, 1708890), with the following modifications: 1 µL iScript Reverse Transcriptase was modified to 0.5 µL. Samples were then subjected to quantitative PCR (qPCR) using the CFX96 Real-Time System (Bio-Rad, New York, NY, USA) with the iTaq™ Universal SYBR^®^ Green Supermix (Bio-Rad, 1725271). The program was set up as follows: initial denaturation at 95 °C for 3 min, then 40 cycles of 95 °C for 15 s (denaturation), and 60 °C for 30 s (annealing and extension). A melting curve was included to test for PCR product purity: 55–95 °C in 0.5 °C increments. *Dram1* primers (Sigma-Aldrich) used are as follows: forward: 5′-CCAGCTTCTTGGTCCGACG-3′; reverse: ‘5-GGGAGAAAGGGGTTGACGTG-3’. *Gapdh* was used as the housekeeping gene: forward: 5′- ATGGTGAAGGTCGGTGTGAA-3′; reverse: ‘5-CTGGAACATGTAGACCATGT-3’.

### 2.7. Western Blot

Cells were harvested, lysed in RIPA buffer (Cell Signalling, Leiden, the Netherlands, 9806) containing a protein inhibitor cocktail (Roche, Basel, Switzerland, 11873580001), and centrifuged at 4 °C for 10 min at 12,000× *g*/min. Western blotting was performed using 15% polyacrylamide gel for LC3 and 10% for polyacrylamide gels for DRAM1 and LAMP1, followed by protein transfer to commercial PVDF membranes (Bio-Rad, 1704156). Membranes were blocked with 3% BSA in 1× Tris-buffered saline (TBS) solution, with 0.1% Tween-20 (TBST), before incubating with primary and secondary antibodies: polyclonal anti-rabbit DRAM1 (1:1000) (Aviva systems biology, San Diego, CA, USA, ARP47432- P050), GAPDH (1:1000)(D16H11), LC3 (1:1000) (Novus Biologicals, NB100-2331), and LAMP1 (1:1000) (Abcam, ab24170), as well as anti-rabbit IgG, and HRP-linked antibody (1:1000) (Cell Signaling, 7074S). Digital images were acquired using the Bio-Rad Universal Hood II imaging system (720BR/01565 UAS). Band intensities were quantified with ImageJ, and values were normalised to GAPDH as a loading control.

### 2.8. Confocal Laser Scanning Microscopy and Analysis of Colocalisation Patterns

RAW 264.7 cells stained by immunofluorescence and/or LysoTracker dye were imaged with a TCS SP8 confocal microscope (Leica) using a 63× oil immersion objective (NA: 1.40). Image processing and the analysis of fluorescent intensity and particles were performed using ImageJ software. Quantification of DRAM1–*Mm* colocalisation involved the characterisation into four categories (none, membrane, punctate, luminal), based on visual stack-by-stack inspection of the confocal Z-stack images. ‘None’: no colocalisation between DRAM1 and *Mm*. ‘Membrane’: DRAM1 staining surrounds *Mm* in a ring. ‘Punctate’: DRAM1 colocalises with *Mm,* but not along *Mm*’s entire length. ‘Luminal’: DRAM1 completely colocalises with *Mm* inside the lumen of a *Mm*-containing vesicle. The same definitions were used to quantify LC3–*Mm* and LAMP1–*Mm* colocalisations, where we note that the membrane pattern was not observed for LC3–*Mm*. *Mm* clusters fully colocalising with LysoTracker were also quantified. For quantification we imaged different regions of interest (ROIs) of the cells that were grown on coverslips in well plates, as described by Green et al. [39]. ROIs were randomly selected by navigating over the slides from left to right and from top to bottom, in order to avoid selecting the same ROI twice. Each ROI contained around 10 cells.

### 2.9. CFU Assay

At 7 h post infection, cells were lysed in water containing 0.05% SDS for 10 min. Lysates of *Mm*-infected cells were serially diluted with 5 steps in 7H9 broth, and 10 µL droplets were spotted onto Middlebrook 7H10 agar plates. The plates were incubated at 32 °C until single colonies were clearly seen, and bacterial colonies were quantified.

### 2.10. SYTOX Green Staining

At 7 h post infection, SYTOX™ Green Nucleic Acid Stain (ThermoFisher Scientific, S7020) was used at a final concentration of 5 μM for 10 min, in order to quantify cell death in different cell lines. Cells were then washed 3 × 5 min before fixation.

### 2.11. Statistical Analyses

In colocalisation studies, the mean probability of colocalisation per experimental condition was estimated, using a generalised linear regression model from the glmmTMB package in R [40]. A beta-binomial family (logit link) was chosen to account for overdispersion when aggregating the number of clusters per region of interest. Post-hoc analysis was performed on the logit scale using Dunnett’s multiple comparison procedure to compare each experimental condition against a single control, by using the emmeans package in R [41]. The mean probabilities per replicate were obtained by taking the inverse of the logit function. Other experiments were analysed by a one-way ANOVA and pairwise comparison with Dunnett’s correction. All graphs were made in GraphPad Prism8 (mean ± SD).

## 3. Results

### 3.1. DRAM1 Colocalises with Acidified Mm-Containing Vesicles

**Figure 1:**
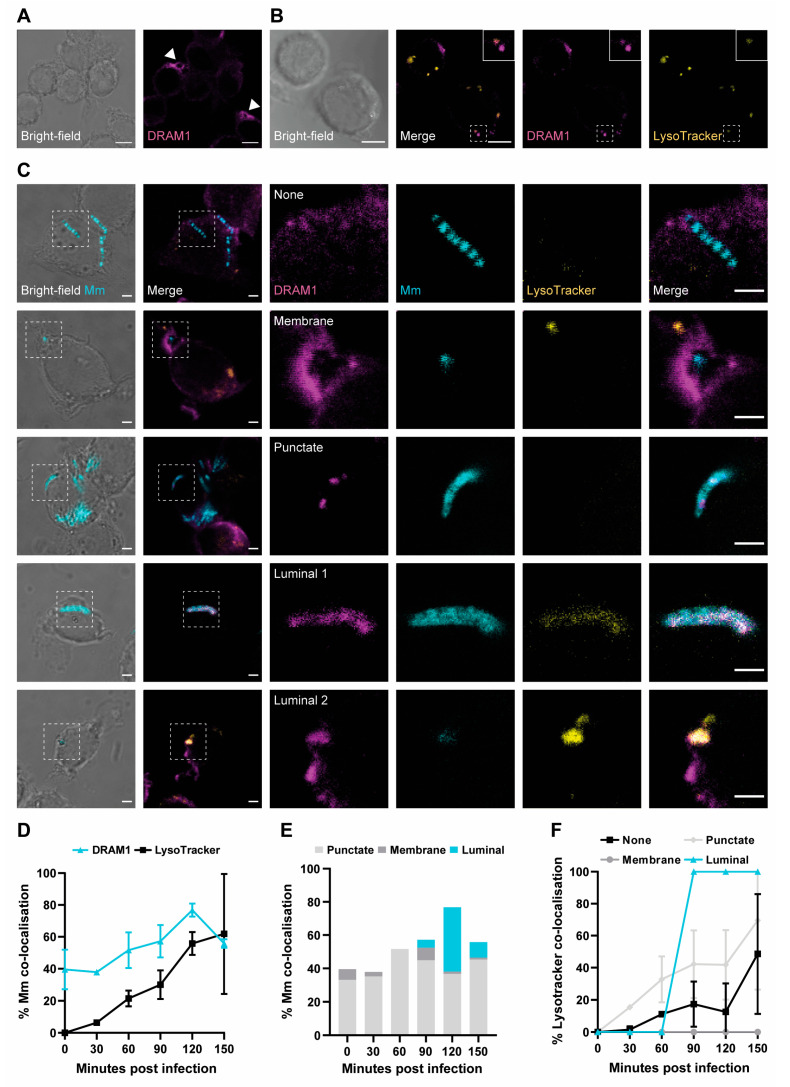
DRAM1 colocalises with acidified mycobacteria-containing vesicles. (**A**) DRAM1 (magenta) localisation at the plasma membrane (arrowheads) of non-infected cells. Scale bars: 5 μm. (**B**) DRAM1 (magenta) colocalisation with acidic vesicles stained by LysoTracker (yellow). A region showing a LysoTracker-positive and a LysoTracker-negative DRAM1-stained vesicle is outlined, with a dotted box in the merged image that is enlarged in the inset. Scale bars: 5 μm. (**C**) Representative examples of DRAM1 (magenta) colocalisation patterns with *Mm* (cyan), referred to as membrane, punctate, and luminal patterns. An example of DRAM1-negative *Mm* (none) is also shown. Acidification of *Mm*-containing vesicles was assessed by LysoTracker staining (yellow). Scale bars: 2 μm. (**D**) Frequency of *Mm* colocalisation with DRAM1 or LysoTracker over time. (**E**) Frequency of *Mm* colocalisation with DRAM1 in punctate, membrane, or luminal patterns over time. (**F**) Frequency of LysoTracker colocalisation with DRAM1-negative *Mm* and DRAM1-positive membrane, punctate, and luminal *Mm* patterns over time. Data were accumulated from two independent experiments with the following total numbers of *Mm* observations per time point: 71 for t = 0, 73 for t = 30, 105 for t = 60, 98 for t = 90, 102 for t = 120 and 111 for t = 150 min.

To study the localisation of DRAM1 in response to *Mm* infection in RAW264.7 macrophages, we performed immunofluorescence staining. We first determined the localisation pattern of DRAM1 in non-infected conditions. We observed that DRAM1 concentrates at certain areas in the vicinity of the plasma membrane. In these highly concentrated regions, DRAM1-positive circular structures of various sizes were occasionally observed (Figure 1A). In addition, we observed that DRAM1 localised to subcellular vesicles. Based on co-staining with LysoTracker, these vesicles were often acidic (Figure 1B), in agreement with the previous identification of DRAM1 as a largely lysosomal protein [24,42]. We then continued to study DRAM1 localisation during *Mm* infection and followed the acidification of *Mm*-containing vesicles by LysoTracker staining. We determined DRAM1–*Mm* colocalisation patterns over a time course of 150 min with 30 min intervals (Figure 1C, Supplementary Figure S2). Approximately 40–80% of *Mm* colocalised with DRAM1 during this time course, with a peak at 120 min (Figure 1D,E). While DRAM1–*Mm* colocalised from the start of the infection, LysoTracker–*Mm* colocalisation sharply increased from low levels to approximately 50% at 120 min (Figure 1D, Supplementary Figure S2). Over the time course, we could distinguish three main categories of the DRAM1 signal, which we refer to as membrane, punctate, and luminal (Figure 1C). The DRAM1 signals classified in the membrane category did not colocalise with *Mm* but surrounded *Mm*-containing vesicles (Figure 1C). This category was the smallest, representing less than 5% of all DRAM1–*Mm* associations at all time points (Figure 1E). Punctate signals showed as small patches of DRAM1 staining that were closely associated with *Mm* (Figure 1C). These punctate patterns constituted 40–60% of all DRAM1–*Mm* colocalisations, with a relatively stable frequency over the time course (Figure 1E). In the luminal category, DRAM1 staining overlapped fully with *Mm* (Figure 1C, luminal 1) or contained a weak *Mm* signal, which might be interpreted as residual staining from degraded bacteria (Figure 1C, luminal 2). These luminal patterns were not detected before 90 min and were most frequent at 120 min, where approximately 30% of DRAM1–*Mm* associations showed this pattern (Figure 1E). We found that the luminal DRAM1 signals were always LysoTracker-positive (Figure 1C,F). *Mm* surrounded by DRAM1 staining (membrane category) was always LysoTracker-negative (Figure 1F). The LysoTracker staining of punctate DRAM1–*Mm* patterns increased over time, similar to DRAM1-negative *Mm* (Figure 1F). In conclusion, the increasing overlap between the DRAM1 signal and bacteria parallels the acidification of *Mm*-containing vesicles.

### 3.2. Acidified Mm-Containing Vesicles Are Positive for the Autophagy Marker LC3

**Figure 2:**
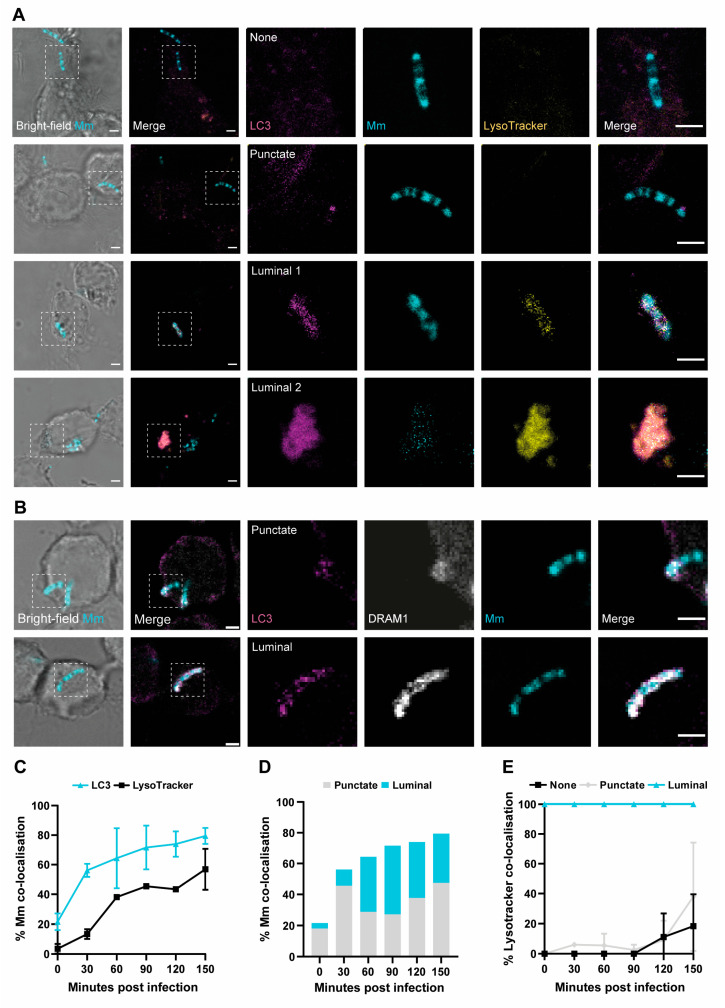
Acidified mycobacteria-containing vesicles are labelled by LC3. (**A**) Representative examples of LC3 (magenta) colocalisation patterns with *Mm* (cyan), referred to as punctate and luminal patterns. An example of LC3-negative *Mm* (none) is also shown. Acidification of *Mm*-containing vesicles was assessed by LysoTracker staining (yellow). Scale bars: 2 μm. (**B**) Representative examples of the double colocalisation of LC3 (magenta) and DRAM1 (grey) with *Mm* (cyan). Scale bars: 2 μm. (**C**) Frequency of *Mm* colocalisation with LC3 or LysoTracker over time. (**D**) Frequency of *Mm* colocalisation with LC3 in punctate or luminal patterns over time. (**E**) Frequency of LysoTracker colocalisation with LC3-negative *Mm* and LC3-positive punctate and luminal *Mm* patterns over time. Data were accumulated from two independent experiments with the following total numbers of *Mm* observations per time point: 99 for t = 0, 129 for t = 30, 96 for t = 60, 124 for t = 90, 133 for t = 120 and 127 for t = 150 min.

Having demonstrated the large overlap between DRAM1 and LysoTracker staining, we wished to further assess the identity of the acidified *Mm*-containing vesicles. Considering that *Mm* can permeabilise phagosomes to invade the cytosol, *Mm* may become a substrate for autophagy. To confirm the cytosolic invasion of *Mm*, we performed immunostaining for ubiquitin, which colocalised both with *Mm* and with LC3, which is a marker for autophagosomes or vesicles formed by LAP (Supplementary Figure S3). Next, we investigated LC3 colocalisation with *Mm* in combination with LysoTracker staining or DRAM1 staining (Figure 2A–C, Supplementary Figure S4). We classified the LC3–*Mm* associations into two categories: punctate and luminal (Figure 2A,D), which were similar to those observed before, in the DRAM1 immunostaining (Figure 1C). In agreement, for both the punctate and the luminal LC3–*Mm* patterns, we observed colocalisation with DRAM1 (Figure 2B). The luminal LC3–*Mm* patterns increased in frequency between 0 and 60 min, after which the punctate and luminal patterns occurred at a relatively stable frequency between 60 and 150 min (Figure 2D). The luminal signals overlapped with either a strong *Mm* signal (Figure 2A, luminal 1), or a weaker, dispersed *Mm* signal (Figure 2A, luminal 2), suggesting that bacterial degradation may have occurred in the latter case, similar to what was observed before in DRAM1 immunostaining (Figure 1C, luminal 2). Quantification showed a nearly complete overlap between luminal LC3 signals and LysoTracker staining, whereas only a subset of punctate LC3 signals was LysoTracker-positive (Figure 2E). In conclusion, LC3 colocalises with acidified *Mm*-containing vesicles in a similar pattern to DRAM1.

### 3.3. DRAM1 Promotes the Acidification of Mm-Containing Vesicles

**Figure 3:**
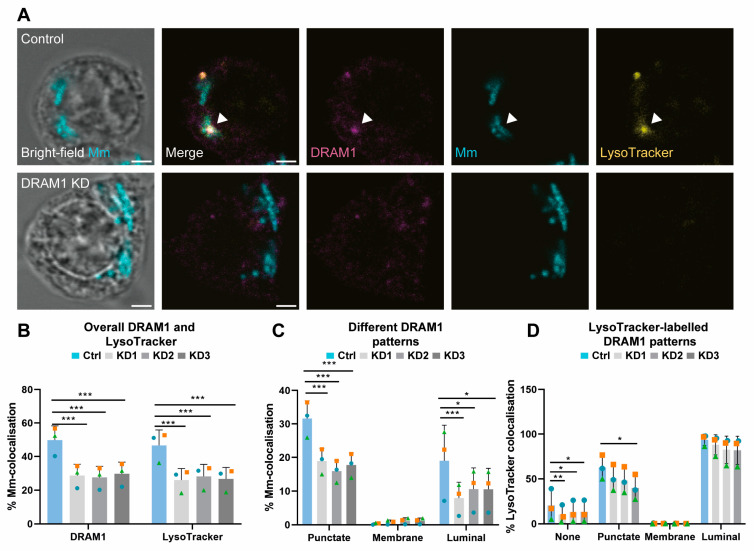
DRAM1 knockdown reduces the acidification of Mm-containing vesicles. (**A**) Representative c with Mm (cyan) in control (Ctrl) and DRAM1 knockdown cell lines (KD1-3), at 120 min post infection. The arrowheads indicate the colocalisation of DRAM1, Mm, and LysoTracker. Scale bars: 2 μm. (**B**) Percentage of colocalisation of Mm with DRAM1 or LysoTracker in DRAM1 knockdown and control cell lines. (**C**) Percentage of colocalisation of Mm with DRAM1 in punctate, membrane, and luminal patterns in DRAM1 knockdown and control cell lines. (**D**) Percentage of colocalisation of LysoTracker with DRAM1-negative Mm (none) and DRAM1-positive punctate, membrane and luminal Mm patterns, in DRAM1 knockdown and control cell lines. Bar graphs show the data from three independent experiments, where the mean of each replicate is indicated with a colored symbol. In total, 18 ROIs were analysed per control or DRAM1 knockdown (KD1-3) group, with the following total numbers of Mm observations per group: Ctrl = 591, KD1 = 632, KD = 525, and KD3 = 567. Statistical significance was assessed by beta-binomial logistic regression with Dunnett’s multiple test correction. Error bars represent the standard deviation of the three independent replications of the experiment. (* *p* < 0.05; ** *p* < 0.01; *** *p* < 0.001).

To study the function of DRAM1 during *Mm* infection in macrophages, we generated three independent DRAM1 shRNA knockdown cell lines by lentiviral transduction, and showed that *Dram1* mRNA expression levels (Supplementary Figure S5A), as well as protein levels (Supplementary Figure S5B), were reduced in each of the three knockdown cell lines compared to the control cells. Next, we analysed the effect of DRAM1 knockdown on *Mm* infection at 120 min post infection, the time point at which DRAM1–*Mm* colocalisation events were most frequent (Figure 1D). Cells were stained for DRAM1 to further validate the knockdown effect, and with LysoTracker to determine the effect of DRAM1 on the acidification of *Mm*-containing vesicles. As expected, the reduced DRAM1 levels due to shRNA knockdown resulted in less DRAM1–*Mm* colocalisation events when compared to the control cells (Figure 3A,B). Furthermore, LysoTracker–*Mm* colocalisation was reduced, consistent with a role for DRAM1 in promoting vesicle acidification (Figure 3A,B). While we still observed the same patterns of colocalisation between DRAM1 and *Mm* in the knockdown cells as in the control, the frequency of luminal patterns was reduced in the knockdown lines (Figure 3C). The colocalisation of these DRAM1–*Mm* patterns with LysoTracker was also affected; specifically, there was a decrease in LysoTracker staining of the punctate DRAM1–*Mm* patterns upon DRAM1 knockdown (Figure 3D). In contrast, the luminal DRAM1–*Mm* patterns almost always remained LysoTracker-positive in the knockdown cell lines. To verify that the decrease in LysoTracker staining of *Mm*-containing vesicles was not caused by an overall reduction in LysoTracker-positive vesicles, due to the knockdown of DRAM1, we performed LysoTracker staining of non-infected cells, which revealed that there were no differences in LysoTracker staining, quantity, or intensity upon DRAM1 knockdown (Supplementary Figure S6A–C). Taken together, DRAM1 knockdown reduced the acidification of *Mm*-containing vesicles with DRAM1-negative or punctate DRAM1 patterns. However, when the remaining DRAM1 protein levels in the knockdown lines led to luminal colocalisation with *Mm*, these events were always associated with acidification.

### 3.4. DRAM1 Mediates LC3 Trafficking to Mm

**Figure 4:**
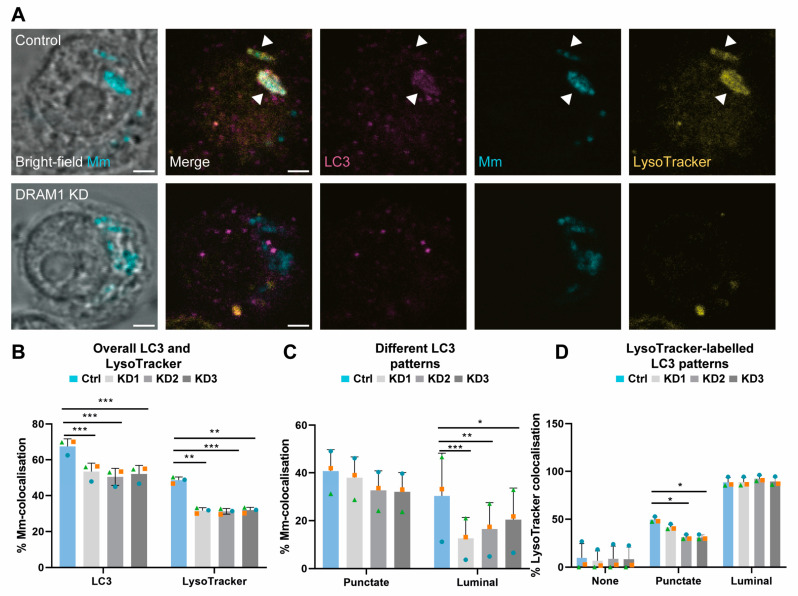
DRAM1 knockdown reduces LC3 trafficking to *Mm*. (**A**) Representative examples of LC3 (magenta) and LysoTracker (yellow) colocalisation with *Mm* (cyan) in DRAM1 knockdown (KD1-3) and control (Ctrl) cell lines at 120 min post infection. The arrowheads indicate the colocalisation of LC3, *Mm* and LysoTracker. Scale bars: 2 μm. (**B**) Percentage of colocalisation of *Mm* with LC3 or LysoTracker in DRAM1 knockdown and control cell lines. (**C**) Percentage of colocalisation of *Mm* with LC3 in punctate and luminal patterns, in DRAM1 knockdown and control cell lines. (**D**) Percentage of colocalisation of LysoTracker with LC3-negative *Mm* and LC3-positive punctate and luminal Mm patterns, in DRAM1 knockdown and control cell lines. Bar graphs show the data from three independent experiments, where the mean of each replicate is indicated with a colored symbol. In total, 18 ROIs were analysed per control or DRAM1 knockdown (KD1-3) group, with the following total numbers of *Mm* observations per group: Ctrl = 527, KD1 = 551, KD2 = 516, KD3 = 537. Statistical significance was assessed by beta-binomial logistic regression with Dunnett’s multiple test correction. Error bars represent the standard deviation of the three independent replications of the experiment. (* *p* < 0.05; ** *p* < 0.01; *** *p* < 0.001).

**Figure 5:**
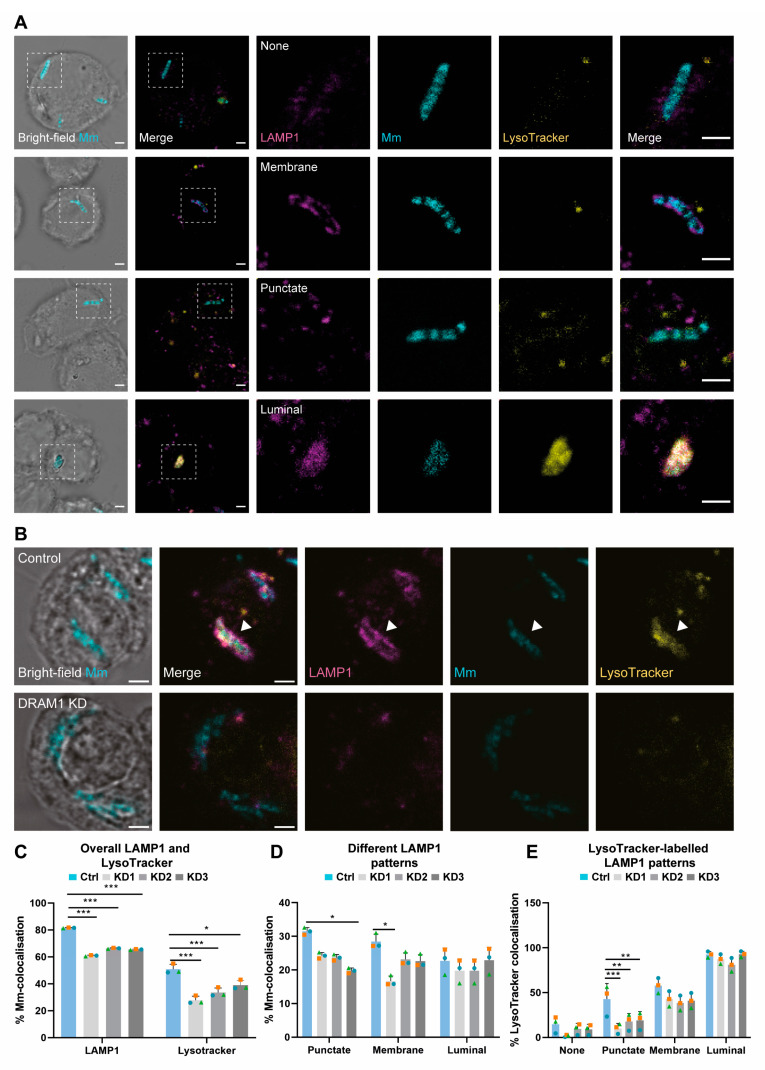
DRAM1 knockdown reduces LAMP1 staining of *Mm*-containing vesicles. (**A**) Representative examples of LAMP1 (magenta) colocalisation patterns with *Mm* (cyan), referred to as membrane, punctate, and luminal patterns, at 120 min post infection. Acidification of *Mm*-containing vesicles was assessed by LysoTracker staining (yellow). Scale bars: 2 μm. (**B**) Representative examples of LAMP1 (magenta) colocalisation with *Mm* (cyan), and of the acidification of *Mm*-containing vesicles, assessed by LysoTracker staining (yellow) in DRAM1 knockdown (KD1-3), as well as control cell lines, at 120 min post infection. The arrowheads indicate colocalisation of LAMP1, *Mm* and LysoTracker. Scale bars: 2 μm. (**C**) Percentage of colocalisation of *Mm* with LAMP1 or LysoTracker in DRAM1 knockdown and control cell lines. (**D**) Percentage of colocalisation of *Mm* with LAMP1 in punctate, membrane, and luminal patterns, in DRAM1 knockdown and control cell lines. (**E**) Percentage of colocalisation of LysoTracker with LAMP1-negative *Mm* and LAMP1-positive *Mm* punctate, membrane, and luminal *Mm* patterns, in DRAM1 knockdown and control cell lines. Bar graphs show the data from three independent experiments, where the mean of each replicate is indicated with a colored symbol. In total, 18 ROIs were analysed per control or DRAM1 knockdown (KD1-3) group, with the following total numbers of *Mm* observations per group: Ctrl = 479, KD1 = 473, KD2 = 469, and KD3 = 502. Significance was assessed by beta-binomial logistic regression with Dunnett’s multiple test correction. Error bars represent the standard deviation of the three independent replications of the experiment (* *p* < 0.05; ** *p* < 0.01; *** *p* < 0.001).

Because we identified that LC3 is recruited to *Mm*-containing vesicles (Figure 2), we next wanted to assess if this step in the vesicle-trafficking process is dependent on DRAM1. We found that the overall percentage of LC3–*Mm* colocalisation was reduced in all knockdown cell lines, similar to the overall percentage of LysoTracker–*Mm* colocalisation (Figure 4A,B). When compared to the control, there was no significant reduction in punctate LC3–*Mm* colocalisation (Figure 4C), though the punctate LC3–*Mm* clusters did display reduced colocalisation with LysoTracker (Figure 4D). DRAM1 deficiency did not reduce basal LC3 levels, as shown by immunostaining and Western blot analysis (Supplementary Figure S6D–F,J); therefore, this could not explain the decreased percentage of colocalisation between *Mm* and LC3. While the punctate LC3–*Mm* colocalisation was not significantly affected, the DRAM1 knockdown cell lines showed a reduction of the luminal LC3–*Mm* colocalisation pattern (Figure 4C). The near-complete colocalisation of luminal LC3–*Mm* patterns with LysoTracker remained unchanged with DRAM1 knockdown (Figure 5D). To conclude, these results support that DRAM1 promotes the trafficking of LC3 to *Mm,* and provide further evidence for the role of DRAM1 in the acidification of *Mm*-containing vesicles.

### 3.5. DRAM1 Promotes the Fusion of LAMP1-Positive Lysosomes with Mm-Containing Vesicles

The reduced acidification of *Mm*-containing vesicles under DRAM1 knockdown conditions prompted us to investigate the role of DRAM1 in mediating the interaction between *Mm* and the lysosomal marker protein LAMP1. Similar to the DRAM1 immunostaining definitions, we could classify LAMP1–*Mm* associations into three patterns: membrane, punctate and luminal (Figure 5A). Consistent with the reduced LysoTracker staining, we observed a reduction in the overall level of LAMP1–*Mm* colocalisation as a result of DRAM1 knockdown (Figure 5B,C). In addition, the membrane and punctate patterns, but not the luminal colocalisation patterns, were reduced in the DRAM1 knockdown cells, as compared to control cells when analysed separately (Figure 5D). DRAM1 knockdown also reduced the colocalisation of punctate LAMP1–*Mm* clusters with LysoTracker, but not for the membrane or luminal patterns (Figure 5E). Based on immunostaining and Western blot analysis of non-infected cells, the effect of DRAM1 knockdown on LAMP1–*Mm* patterns is not caused by a general reduction in LAMP1 protein levels (Supplementary Figure S6G–I,K). In conclusion, the effect of DRAM1 knockdown on the overall number of LAMP1–*Mm* colocalisation events and the reduced LysoTracker staining of punctate LAMP1–*Mm* patterns is consistent with a role for DRAM1 in mediating the interaction of lysosomes with *Mm*-containing vesicles.

### 3.6. DRAM1 Is Required for Macrophage Defence against Mm

**Figure 6:**
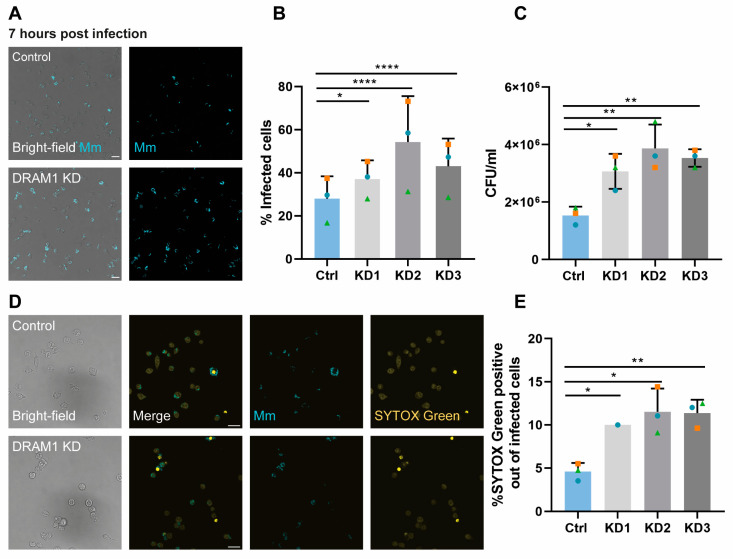
DRAM1 knockdown increases the susceptibility of macrophages to *Mm* infection. (**A**) Representative examples of infected cells in DRAM1 knockdown and control cell lines at 7 h post infection. Scale bars: 20 μm (**B**) Percentage of infected cells in DRAM1 knockdown (KD1-3) and control cells at 7 h post infection. (**C**) CFU/mL in DRAM1 knockdown cells normalised to the control cell lines at 7 h post infection. (**D**) Representative examples of SYTOX Green staining of *Mm*- infected DRAM1 knockdown and control cells at 7 h post infection. Scale bars: 20 μm. (**E**) Percentage of SYTOX green-positive infected cells in DRAM1 knockdown and control cell lines at 7 h post infection. Bar graphs (**B**,**C**,**E**) show the data from three independent experiments, where the mean of each replicate is indicated with a colored symbol. All replicates in (**B**,**C**) were performed with 3 knockdown lines (KD1-3), while the data in (**E**) are based on 3 replicates for KD2 and KD3 and 1 replicate for KD1. Statistical significance was assessed by one-way ANOVA and pairwise comparison with Dunnett’s correction. (* *p* < 0.05; ** *p* < 0.01; **** *p* < 0.0001).

Having determined the requirements of DRAM1 for *Mm*’s colocalisation with LysoTracker, LC3 and LAMP1, we wished to understand whether this role of DRAM1 in *Mm* vesicle trafficking impacts the susceptibility of RAW 264.7 macrophages to *Mm* infection. DRAM1 knockdown cell lines were infected with *Mm* for 1 h, in order to understand whether DRAM1 affected *Mm* phagocytosis. The results showed no significant difference in the percentage of infected cells between the knockdown and control groups, indicating that DRAM1 does not impair the ability of RAW264.7 macrophages to phagocytose *Mm* (Supplementary Figure S7A,B). Next, to determine if DRAM1 reduces *Mm* burden, cells were infected for a total of 7 h. Results showed that the percentage of infected cells in DRAM1 knockdown cell lines was higher than in the control group at 7 h post-infection (Figure 6A,B), indicating that DRAM1 protects against *Mm* infection without affecting phagocytosis in RAW 264.7 macrophages. In agreement, bacterial colony-forming unit (CFU) counts from DRAM1 knockdown cells were higher than in the control group (Figure 6C). Moreover, SYTOX Green staining revealed increased levels of cell death in infected DRAM1 knockdown cells (Figure 6D,E). Therefore, we conclude that DRAM1 loss-of-function impairs the ability of macrophages to control *Mm* infection.

## 4. Discussion

Understanding the mechanisms that deliver pathogens to lysosomes for degradation is crucial for developing novel therapeutic interventions against pathogens, such as mycobacteria, that are able to counteract both the phagolysosomal pathway and autophagy [43]. Based on studies in the zebrafish model, DRAM1 has emerged as a host resistance factor that promotes the delivery of mycobacteria to autophagosomes and lysosomes [20,30]. Here we confirm and extend these findings in a mammalian model, demonstrating the colocalisation that occurs between DRAM1 and mycobacteria in the context of LC3-labelled and lysosomal compartments during the course of *Mm* infection in RAW264.7 macrophages. Furthermore, we show that DRAM1 knockdown impairs the macrophage defence response against *Mm* infection. These results support the central role of DRAM1 in the lysosomal delivery of mycobacteria.

We have previously detected DRAM1 colocalisation with *Mtb* in primary human macrophages, but this cell type did not permit efficient DRAM1 knockdown studies [20]. Knockdown, as well as loss-of-function mutation, analyses of zebrafish DRAM1 supported its requirement for autophagic defence against *Mm* [20,30]. However, a detailed analysis of DRAM1–mycobacteria colocalisation as well as a functional study in a mammalian cell type were still lacking. Therefore, we now employed an *Mm* infection model in murine RAW 264.7 macrophages. In uninfected RAW 264.7 cells, we detected DRAM1 on lysosomes, which is consistent with studies in other cell types that identified DRAM1 and its splice variants as being largely endosomal/lysosomal proteins, although they are also present also on autophagosomes, the endoplasmic reticulum and peroxisomes [24,42]. In addition, we found that DRAM1 was prominently expressed in regions at or near the plasma membrane of RAW264.7 macrophages. Plasma membrane expression of DRAM1 has also been observed in hepatocytes, and two other members of the DRAM family, DRAM3 and DRAM5, also display plasma membrane expression [27,29,44]. Additionally, we observed the formation of circular DRAM1-positive structures near the plasma membrane that might be the result of endocytosis, for example, of cell debris, and that, therefore, derived from the plasma membrane in regions where DRAM1 is highly concentrated. This expression pattern of DRAM1 is particularly interesting in the light of a recent study reporting a role for DRAM1 in extracellular vesicle formation [44]. Since exosomes are crucial for communication between immune cells, this might point to an unexplored role for DRAM1 in the intercellular propagating of signals during infection.

In the present work we focused on the role of DRAM1 in intracellular host defence. During *Mm* infection, we found that the DRAM1 signal occasionally enveloped mycobacteria in a circular pattern, but most frequently appeared in a punctate pattern closely associated with mycobacteria, which gradually progressed to a full colocalisation pattern. The progression from partial to full colocalisation was concomitant with DRAM1–LC3–mycobacteria colocalisation, as well as DRAM1–LysoTracker–mycobacteria colocalisation and DRAM1–LAMP1–mycobacteria colocalisation, which strengthens the previous functional studies in zebrafish that have linked DRAM1 to the trafficking of mycobacteria along the (auto)phagolysosomal pathway [20,30]. Based on the colocalisation patterns and their frequency over time, we propose the following sequence of events. After phagocytosis, *Mm* first resides in a DRAM1-negative non-acidic phagosome, possibly recruiting LC3 through LAP. Subsequently DRAM1 may be recruited to the phagosomal membrane. Lysosomes containing DRAM1 within the membrane may then fuse with the *Mm*-containing vesicle, leading to its acidification. Alternatively, *Mm* escapes from the phagosome into the cytoplasm and is subsequently trapped in an autophagosome. This scenario is supported by the observation that *Mm* in RAW264.7 macrophages is ubiquitinated, making it a substrate for ubiquitin receptor-mediated xenophagy. The continued recruitment of DRAM1 to *Mm*-containing vesicles, of either a phagosomal or autophagosomal nature, would result in an increase in partial DRAM1–mycobacteria colocalisation. Subsequently, we hypothesise that DRAM1 becomes luminal following the fusion of phagosomes or autophagosomes with lysosomes, resulting in full DRAM1–mycobacteria colocalisation in the acidic and degradative environment of autolysosomes and endolysosomes. Another potential explanation for the full colocalisation pattern is that it is derived from the invagination of the membrane enclosing *Mm*, resulting in the formation of multi-vesicular bodies (MVBs) that contain intraluminal vesicles with DRAM1 in the membrane. This would imply that lysosomes do not fuse until after complete DRAM1–mycobacteria colocalisation, as MVBs are formed at a late stage of vesicle maturation, before lysosome fusion [45].

Previously, DRAM1 was shown to regulate autophagic flux through the lysosomal v-ATPase in human A549 cells, under conditions of mitochondrial stress [46]. To determine DRAM1′s function in the (auto)phagolysosomal pathway of infected macrophages, we generated shRNA knockdown RAW 264.7 cell lines. DRAM1 knockdown led to reduced associations with LC3, as well as reduced acidification of *Mm*-containing vesicles, suggesting that DRAM1 mediates autophagic targeting and acidic vesicle trafficking to *Mm*. This reflects results seen in zebrafish, where DRAM1 knockdown or mutations resulted in reduced GFP–LC3-*Mm* colocalisation and reduced LysoTracker–*Mm* colocalisation [20,30]. Extending from this, our analysis in RAW 264.7 macrophages demonstrated that there was a reduced acidification of *Mm*-containing compartments showing partial or no colocalisation with the remaining DRAM1 protein in the knockdown lines. In contrast, despite DRAM1 knockdown, *Mm*-containing vesicles that showed full colocalisation with DRAM1 remained acidic. This suggests that DRAM1 affects the trafficking of acidic vesicles to *Mm*-containing vesicles, primarily at the early stages of vesicle maturation. In agreement, the effect of DRAM1 knockdown on LysoTracker–*Mm* colocalisation was larger than the effect on LAMP1–*Mm* colocalisation, suggesting that DRAM1 knockdown affects not only the fusion of lysosomes with *Mm*-containing vesicles, but also the acidification of these vesicles, due to fusion with late endosomes at an earlier step in the maturation process. The proposed role of DRAM1 in promoting fusion between acidic vesicles and *Mm*-containing compartments is supported by results observed in zebrafish overexpressing DRAM1, where large vesicles containing membrane remnants were observed in transmission electron micrographs, suggesting that DRAM1 mediates multiple vesicle fusion events [20]. The stimulatory action of DRAM1 on vesicle trafficking and fusion in the (auto)phagolysosomal pathway likely enhances the microbicidal activity against mycobacteria, as we observed that DRAM1 knockdown led to an overall increased *Mm* infection burden in RAW 264.7 macrophages.

## 5. Conclusions

The results of this study, which show the requirement of DRAM1 for (auto)phagolysosomal delivery of *Mm* in RAW 264.7 macrophages, support the role of DRAM1 as a host resistance factor that is conserved across zebrafish and mammals. Therefore, DRAM1 represents a putative target for host-directed anti-mycobacterial therapy that could help overcome current challenges posed by anti-microbial resistance. Therapeutic strategies targeting DRAM1 might be more broadly applicable against other intracellular pathogens, considering our recent results that DRAM1 also protects against infections with *Salmonella enterica* serovar Typhimurium and *Aspergillus fumigatus* [47,48]. Further elucidating its mechanism of action will facilitate the development of therapeutic strategies that exploit the role of DRAM1 in vesicle trafficking and fusion in the (auto)phagolysosomal pathway.

## Data Availability

Data available in a publicly accessible repository: 10.5281/zenodo.7701960.

## Supplementary Materials

The following supporting information can be downloaded at: https://www.mdpi.com/article/10.3390/cells12060828/s1.
