# Supplementary material for: DRAM1 Promotes Lysosomal Delivery of Mycobacterium marinum in Macrophages
Source: Cells. 2023 Mar 7;12(6):828. doi: 10.3390/cells12060828 (PMC10047064; doi:10.3390/cells12060828)
Supplement: Supplementary file 1 [file cells-12-00828-s001.zip › cells-2012823-supplementary.pdf]

cells

MDPI

Article

DRAM1 Promotes Lysosomal Delivery of *Mycobacterium marinum* in Macrophages

Adrianna Banducci-Karp †, Jiajun Xie †, Sem A. G. Engels, Christos Sarantaris, Patrick van Hage, Monica Varela, Annemarie H. Meijer *,‡ and Michiel van der Vaart *,‡

Institute of Biology Leiden, Leiden University, Einsteinweg 55, 2333 CC Leiden, The Netherlands
* Correspondence: a.h.meijer@biology.leidenuniv.nl (A.H.M.); m.vandervaart@hartstichting.nl (M.v.d.V.)
† These authors contributed equally to this work.
‡ These authors contributed equally to this work.

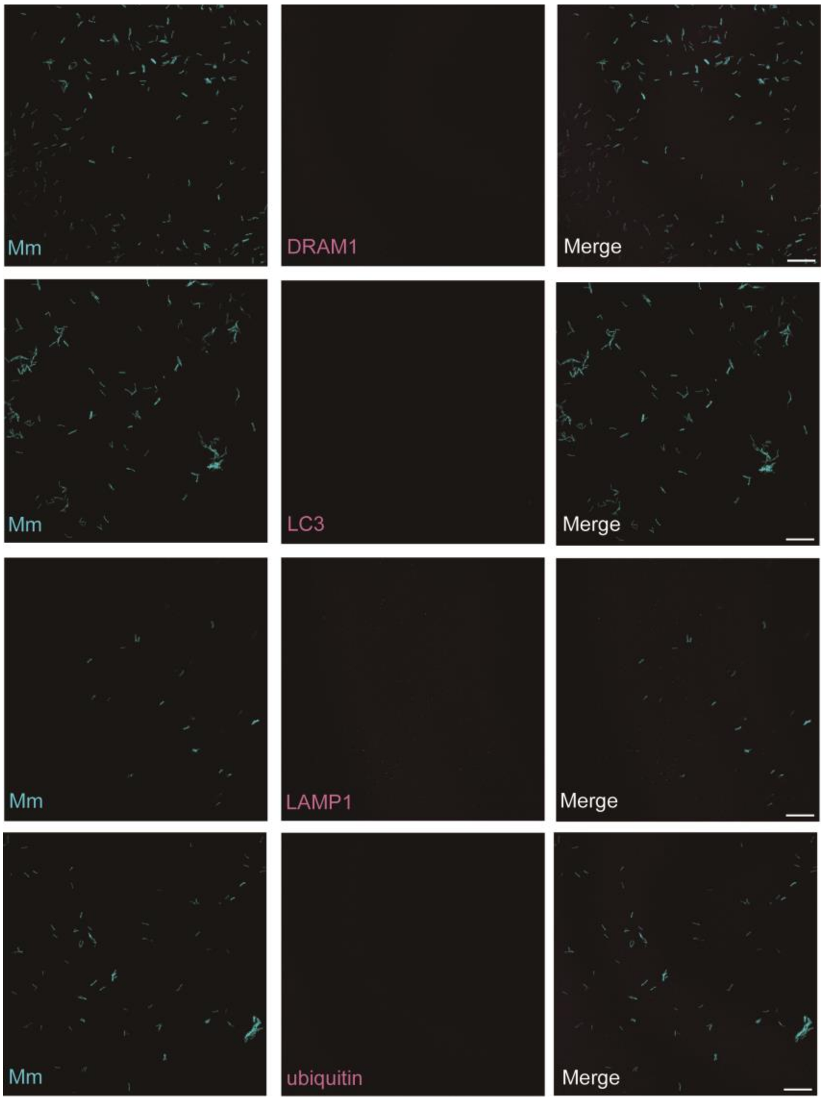

Figure S1. Lack of cross-reaction of antibodies with in vitro cultured *Mm*. Green-fluorescent *Mm* bacteria were cultured directly onto slides and fixed for immunostaining by DRAM1, LC3, LAMP1 and ubiquitin primary antibodies, followed by detection with goat-anti-rabbit Alexa Fluor™ Plus 647 secondary antibody. The same conditions were used as for immunostaining of *Mm*-infected RAW264.7 macrophages. No overlap of antibody signals with *Mm* were detected. Scale bars: 20 μm.

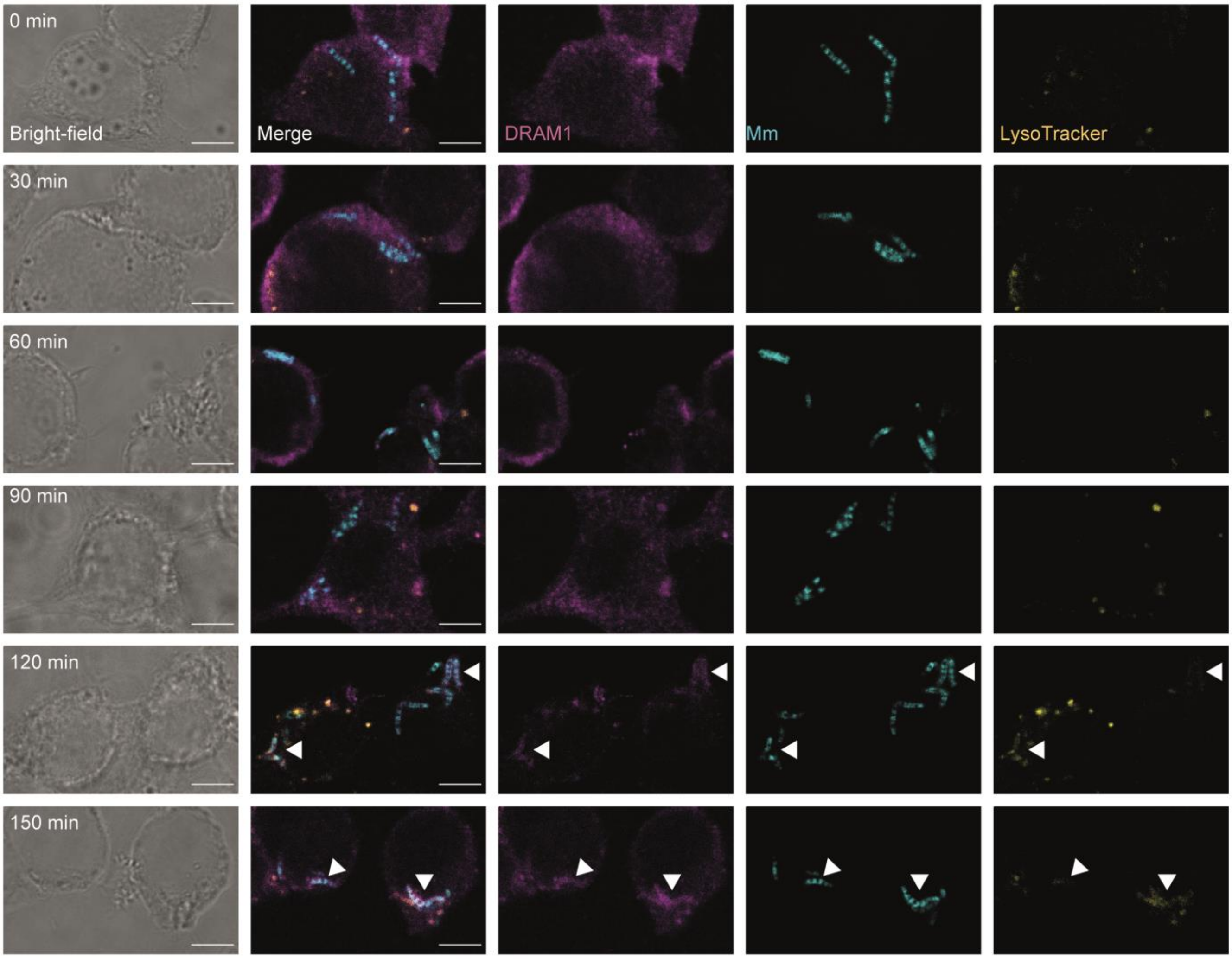

Figure S2. Representative examples of DRAM1 (magenta) colocalisation with LysoTracker (yellow) and *Mm* (cyan) from 0 to 150 min post infection. The arrowheads indicate colocalisation of luminal DRAM1, *Mm* and LysoTracker. Scale bars: 5 µm.

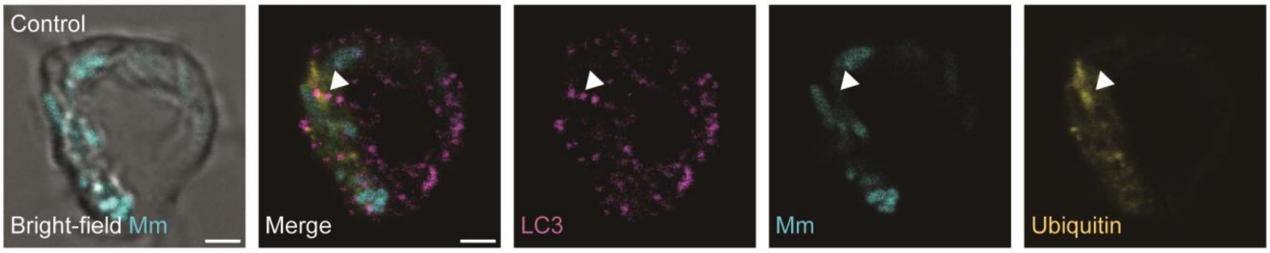

Figure S3. Representative example of ubiquitin staining (yellow) of *Mm* (cyan) and colocalisation with LC3 (magenta), indicated with an arrowhead, at 120 min post infection. Scale bars: 2 μm.

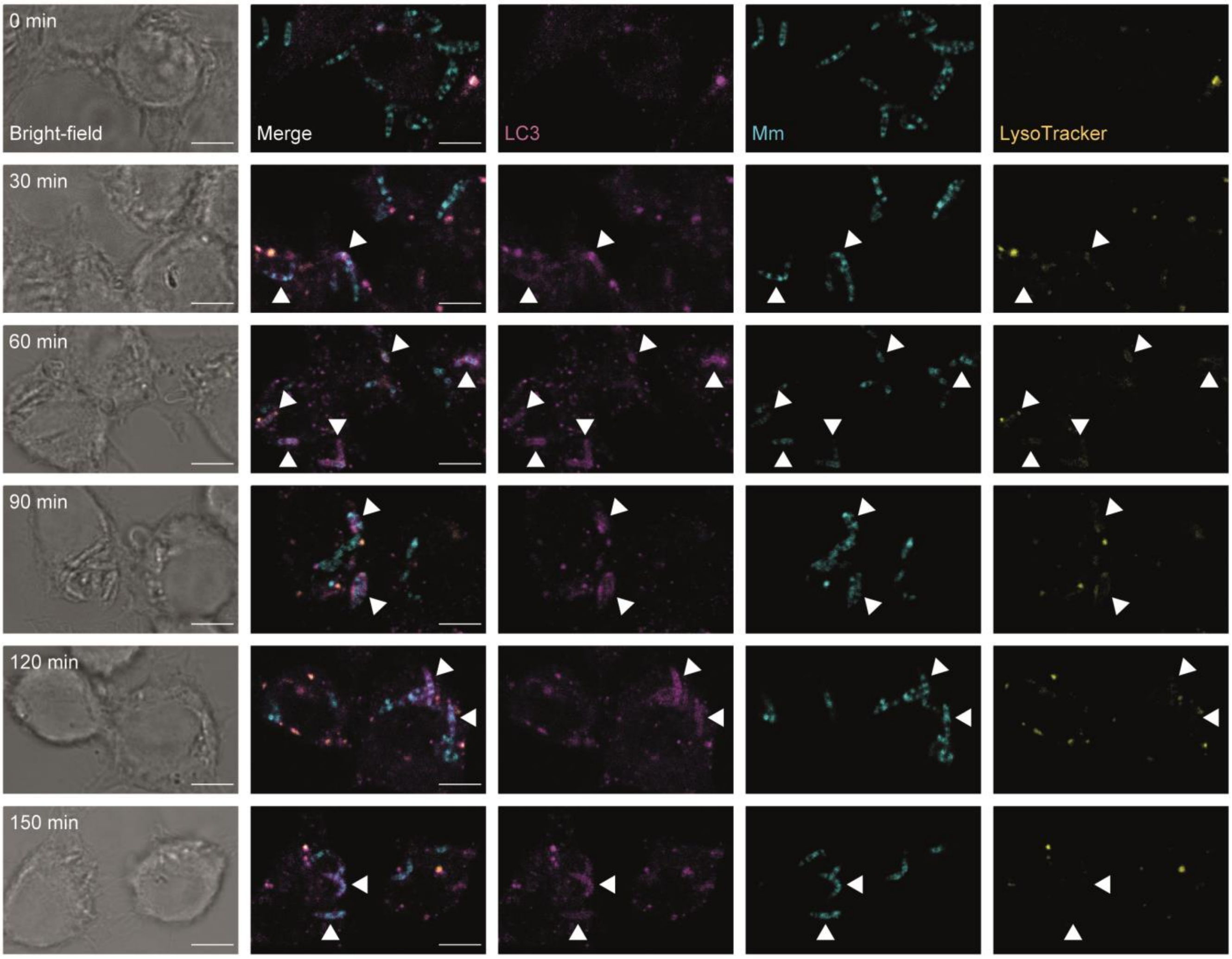

Figure S4. Representative examples of LC3 (magenta) colocalisation with LysoTracker (yellow) and *Mm* (cyan) from 0 to 150 min post infection. The arrowheads indicate colocalisation of luminal LC3, *Mm* and LysoTracker. Scale bars: 5 µm.

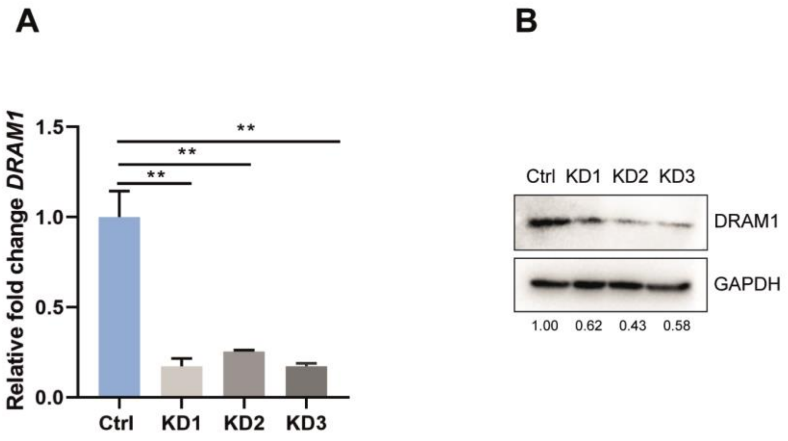

Figure S5. Confirmation of DRAM1 knockdown in RAW 264.7 macrophages. (A) qPCR analysis of *Dram1* expression levels in control and knockdown cell lines (KD1-3). Statistical significance is assessed by one-way ANOVA and pairwise comparison with Dunnett's correction. (**p<0.01) (B). Western blot analysis of DRAM1 protein levels in control and knockdown cells. Each cell line is analyzed in duplicate. DRAM1 protein levels were quantified relative to the control and normalized to GAPDH and are indicated underneath the western blot.

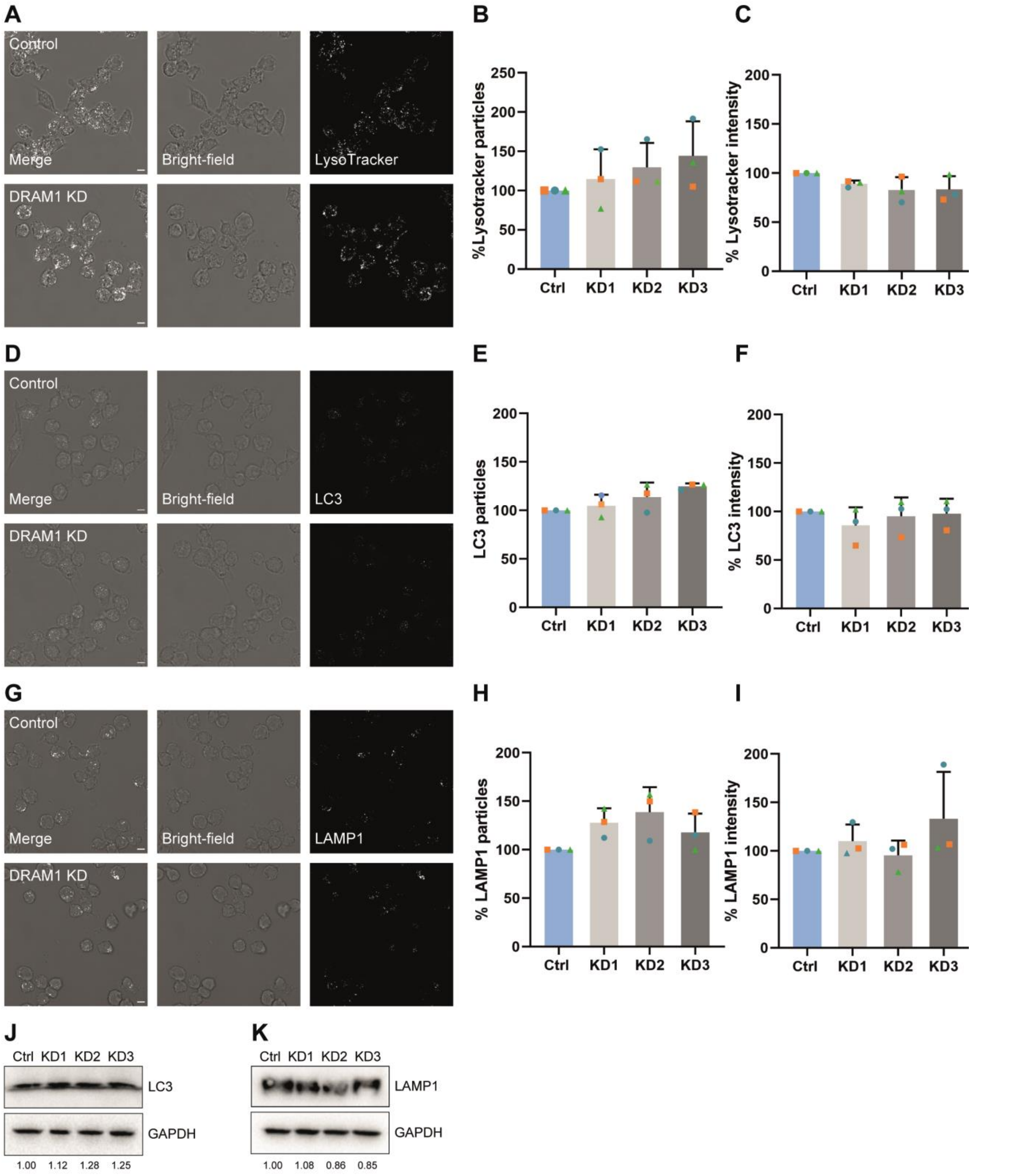

Figure S6. DRAM1 knockdown does not affect overall levels of LysoTracker, LC3 and LAMP1 (A,D,G) Representative examples of LysoTracker (A), LC3 (D) and LAMP1 (G) staining in non-infected DRAM1 knockdown (KD1-3) and control cells. Scale bars: 5 μm. (B,C,E,F,H,I) Relative quantification of LysoTracker (B,C), LC3 (E,F) and LAMP1 (H,I) particles and signal intensity in non-infected DRAM1 knockdown and control cells. (J,K) Western blot analysis of LC3 (J) and LAMP1 (K) protein level relative to GAPDH in non-infected DRAM1 knockdown and control cells. Bar graphs (B,C,E,F,H,I) show the data from three independent experiments (36 ROIs), where the mean of each replicate is indicated with a colored symbol. Particles/intensity analysis are normalized by number of cells. Statistical significance is assessed by one-way ANOVA with Dunnett's correction.

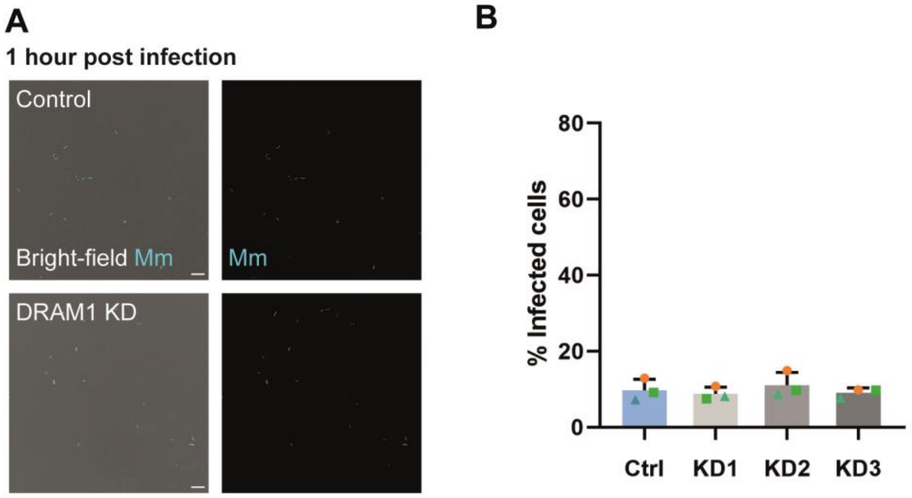

Figure S7. DRAM1 knockdown does not affect phagocytosis of *Mm* in RAW264.7 macrophages. (A) Representative examples of infected cells in DRAM1 knockdown and control cells at 1 h post infection. (B) Percentage of infected cells in DRAM1 knockdown (KD1-3) and control cells at 1 h post infection. Scale bars: 20 μm. Bar graphs show the data from three independent experiments (36 ROIs), where the mean of each replicate is indicated with a colored symbol. Statistical significance is assessed by one-way ANOVA and pairwise comparison with Dunnett's correction.
